# Germline *PALB2* Genetic Variant Associated with Rapid Metastatic Progression and Poor Survival in Two Kazakh Women with Breast Cancer: A Case Study

**DOI:** 10.3390/genes17080913

**Published:** 2026-07-31

**Authors:** Gulnur Zhunussova, Nazgul Omarbayeva, Aigul Zhunussova, Diana Abdullayeva, Liliya Skvortsova, Nursultan Nurdinov, Ainash Oshibayeva

**Affiliations:** 1 Laboratory of Molecular Genetics, Institute of Genetics and Physiology, Almaty 050060, Kazakhstan; aigul.s.zhunussova@gmail.com (A.Z.); lilia_555@rambler.ru (L.S.); 2 Oncology Department, Asfendiyarov Kazakh National Medical University, Almaty 050012, Kazakhstan; nazgul_omarbayeva@mail.ru (N.O.); d_abd2003@mail.ru (D.A.); 3 Department of Mammology, Kazakh Institute of Oncology and Radiology, Almaty 050022, Kazakhstan; 4 Department of Fundamental Medical Disciplines, Faculty of Dentistry, Khoja Akhmet Yassawi International Kazakh-Turkish University, Turkestan 161200, Kazakhstan; nursultan.nurdinov@ayu.edu.kz; 5 Department of Public Health and Scientific Research, Faculty of Medicine, Khoja Akhmet Yassawi International Kazakh-Turkish University, Turkestan 161200, Kazakhstan; ainash.oshibayeva@ayu.edu.kz

**Keywords:** breast cancer, *PALB2*, germline variant, hereditary breast cancer, aggressive disease progression, Kazakhstan

## Abstract

**Background**: Germline *PALB2* variants are associated with hereditary breast cancer risk, but their clinical impact in Central Asian populations remains largely uncharacterized. This case study aims to evaluate the clinical significance of *PALB2* variants in two Kazakh women with early-onset breast cancer. **Methods**: Molecular genetic testing identified germline *PALB2* pathogenic variants (NM_024675.4:c.18_22delGAAGC and NM_024675.4:c.1034T>G) in two Kazakh women with early-onset invasive ductal carcinoma. Clinical courses, treatment responses, and outcomes were followed. **Results**: Neither patient had a reported family history of breast or other malignancies. Patient 1, a 26-year-old pregnant woman, was diagnosed with stage IIIB luminal B, HER2-negative invasive ductal carcinoma and received neoadjuvant chemotherapy, radical surgery, radiotherapy, endocrine therapy, and subsequent treatment for metastatic disease. Despite an initial response, she developed extensive skeletal metastases and died from metastatic breast cancer. Patient 2, a 33-year-old woman, presented with de novo stage IV luminal B, HER2-negative invasive ductal carcinoma with hepatic metastases. Following multimodal treatment, including chemotherapy, surgery, radiotherapy, endocrine suppression, and systemic therapy for disease progression, she experienced further metastatic spread and ultimately died from breast cancer-related complications. **Conclusions**: Both patients exhibited aggressive clinical courses characterized by early disease onset, metastatic progression, and poor outcomes despite comprehensive treatment. These cases highlight the potential clinical significance of germline *PALB2* variants in apparently sporadic breast cancer and underscore the importance of genetic testing, risk assessment, and genetic counselling in young breast cancer patients, particularly in underrepresented.

## 1. Introduction

Cancer is a complex group of diseases characterized by the uncontrolled growth and proliferation of abnormal cells that can invade surrounding tissues and spread to other parts of the body through metastasis [1]. It develops as a result of genetic pathogenic variants [2], and molecular alterations caused by various factors, including environmental exposure [3], unhealthy lifestyle [4], radiation [5], hormonal imbalance [6], aging [7], infectious agents [8], and hereditary predisposition [9].

Among the various types of cancer, breast cancer is considered one of the most significant due to its high incidence and mortality rates among women [10]. Breast cancer is influenced by numerous risk factors, among which hormonal imbalance involving estrogen and progesterone plays a critical role in disease initiation and progression [11]. These steroid hormones have been extensively investigated because of their fundamental involvement in breast physiology and tumorigenesis [6]. Estrogen, primarily synthesized in the ovaries, is essential for the growth, differentiation, and maintenance of breast tissue [12]. Likewise, progesterone regulates mammary gland development and reproductive function [13]. However, disruption of the tightly regulated balance between these hormones can trigger abnormal cellular proliferation and promote breast carcinogenesis [14].

In addition to hormonal influences, inherited pathogenic variants in cancer susceptibility genes play a major role in breast cancer development [15]. *BRCA1* and *BRCA2* are the well-established high-penetrance susceptibility genes [16]. Several other genes have been implicated in hereditary breast cancer predisposition, including *TP53*, *PTEN*, *CDH1*, *STK11*, *CHEK2*, *ATM* and *PALB2*, with varying levels of penetrance and clinical significance. These genes are involved in key cellular processes such as DNA damage repair, cell cycle regulation, genomic stability and apoptosis, and their dysfunction may contribute to tumour initiation, progression and therapeutic response [17].

Among these susceptibility genes, *PALB2* (partner and localizer of BRCA2) has emerged as an important breast cancer predisposition gene because of its central role in homologus recombination mediated DNA repair. *PALB2* acts as molecular bridge between *BRCA1* and *BRCA2*, thereby facilitating the accurate repair of DNA double-strand breaks. Germline pathogenic variants in *PALB2* are associated with a substantially increased risk of breast cancer and are increasingly recognized in both hereditary and apparently sporadic cases.

Beyond cancer predisposition, accumulating evidence indicates that germline *PALB2* pathogenic variants may be associated with more aggressive disease characteristics and unfavourable clinical outcomes. Several studies have reported early-onset disease, advanced-stage presentation, increased metastatic potential, and reduced survival among carriers of pathogenic *PALB2* variants [9]. Furthermore, the identification of *PALB2* variants has important clinical implications for genetic counselling, risk assessment, family screening, and the selection of targeted therapeutic strategies.

Despite increasing recognition of the clinical importance of *PALB2*, data from Central Asian populations remain limited. In Kazakhstan, the contribution of *PALB2* variants to breast cancer susceptibility and disease progression has not been extensively characterized. Consequently, reports describing the clinical manifestations and outcomes of *PALB2*-associated breast cancer in Kazakh patients are scarce.

Here, we present two cases of young Kazakh women with luminal B, HER2-negative invasive ductal breast carcinoma carrying germline *PALB2* variants (NM_024675.4:c.18_22delGAAGC and NM_024675.4:c.1034T>G). Notably, neither patient had a reported family history of breast or other malignancies. Both patients experienced rapid metastatic progression and poor survival despite receiving comprehensive multimodal treatment. These cases highlight the potential clinical significance of germline *PALB2* variants in apparently sporadic breast cancer and emphasize the need for broader implementation of genetic testing and counseling among young breast cancer patients in Kazakhstan.

## 2. Materials and Methods

### 2.1. Sample Collection

Blood samples were collected at the Institute of Oncology and Radiology in Almaty. The KazIOR Ethics Committee accepted the study protocol, which was carried out in compliance with the Declaration of Helsinki. Each participant donated five milliliters of blood for molecular genetic analysis and signed an informed consent form. Additionally, the patients agreed to the anonymous publication of their clinical and molecular-genetic data under a unique ID code. Within hours of collection, collected blood in EDTA tubes was carried in a portable refrigerated container to the Institute of Genetics and Physiology and stored at −20 °C for additional molecular-genetic research. The ordering physician asked each patient about their family history, clinicopathology, and sociodemographic status.

### 2.2. DNA Library Preparation for Parallel Sequencing (NGS) and Data Processing

TruSight Rapid Capture Kit (Illumina Inc., San Diego, CA, USA) was used for DNA Library preparation and concentration was measured with Qubit 2.0 Fluorometer (Invitrogen, Thermo Fisher Scientific, Waltham, MA, USA). Targeted sequencing was performed using the Illumina MiSeq platform (Illumina Inc., San Diego, CA, USA), covering more than 1700 exons and adjacent intronic regions (±50 bp) across 94 cancer-associated genes, encompassing approximately 255 kb of the human genome. Libraries were prepared at a final concentration of 4 nM, cluster-generated on MiSeq flow cells, and sequenced using the MiSeq Reagent Kit v3 (Illumina Inc., San Diego, CA, USA) with 600-cycle paired-end.

Sequencing data were processed using MiSeq Reporter v3.0 (Illumina Inc., San Diego, CA, USA). Reads were aligned to the hg19 (GRCh37.p13) reference genome using BWA v0.7.17, and variants were called with GATK v4.5. Variants with read depth ≤ 50× were excluded, and quality filtering was performed using a minimum Q-score of 30, total read depth > 50×, alternative allele depth > 20×, and variant quality score > 100. Variant annotation was performed using Variant Studio v3.0 (Illumina Inc., San Diego, CA, USA), with interpretation based on ClinVar, dbSNP, LOVD, population databases (1000 Genomes, gnomAD, ExAC, and TOPMed), and published literature. Variants were classified according to ACMG/AMP guidelines [18]. Variants absent from major databases and literature were considered novel.

### 2.3. Validation of Pathogenic Variants Using the Standard Sanger Method

Direct Sanger sequencing was used to validate genetic alterations in *PALB2* gene identified using high-throughput sequencing (NGS) on the MiSeq platform (Illumina, San Diego, CA, USA) and the TruSight Rapid Capture kit (Illumina, San Diego, CA, USA). Specific primers were designed and synthesized using NCBI Primer Blast (National Center for Biotechnology Information, Bethesda, MD, USA), Primer3web version 4.1.0, and IDT OligoAnalyser (Integrated DNA Technologies, Coralville, IA, USA). A Mastercycler nexus thermocycler (Eppendorf SE, Hamburg, Germany) and specific primers were used for PCR amplification of the gene fragments. Primer sequences and PCR details are presented in Table 1. PCR products were sequenced on an ABI 3500XL Genetic Analyzer (Applied Biosystems, Foster City, CA, USA) using the BigDye™ Terminator v3.1 Cycle Sequencing Kit (Applied Biosystems, Foster City, CA, USA) and purified with the BigDye XTerminator™ Purification Kit (Applied Biosystems, Foster City, CA, USA) according to the manufacturer’s instructions. Sequence chromatograms were analyzed using Chromas v2.6.6 software (Technelysium Pty Ltd., South Brisbane, QLD, Australia).

### 2.4. Genotyping of Pathogenic Variants by PCR-RFLP

Genotyping of pathogenic variants c.1034T>G and c.18_22del in the *PALB2* gene is carried out by the polymerase chain reaction method followed by restriction of amplifications and restriction fragment length analysis (PCR-RFLP). The PCR products were digested at 37 °C for 8–16 h with 1–3 U of the corresponding restriction enzymes (New England Biolabs, Ipswich, MA, USA) and analyzed on 2% agarose gel.

Genotypes (mutant and wild type) of pathogenic variants can be distinguished by the length of fragments after restriction of amplicons (Table 2).

## 3. Results

### 3.1. PALB2 Variants Identified in Two Young Breast Cancer Patients

**Figure 1:**
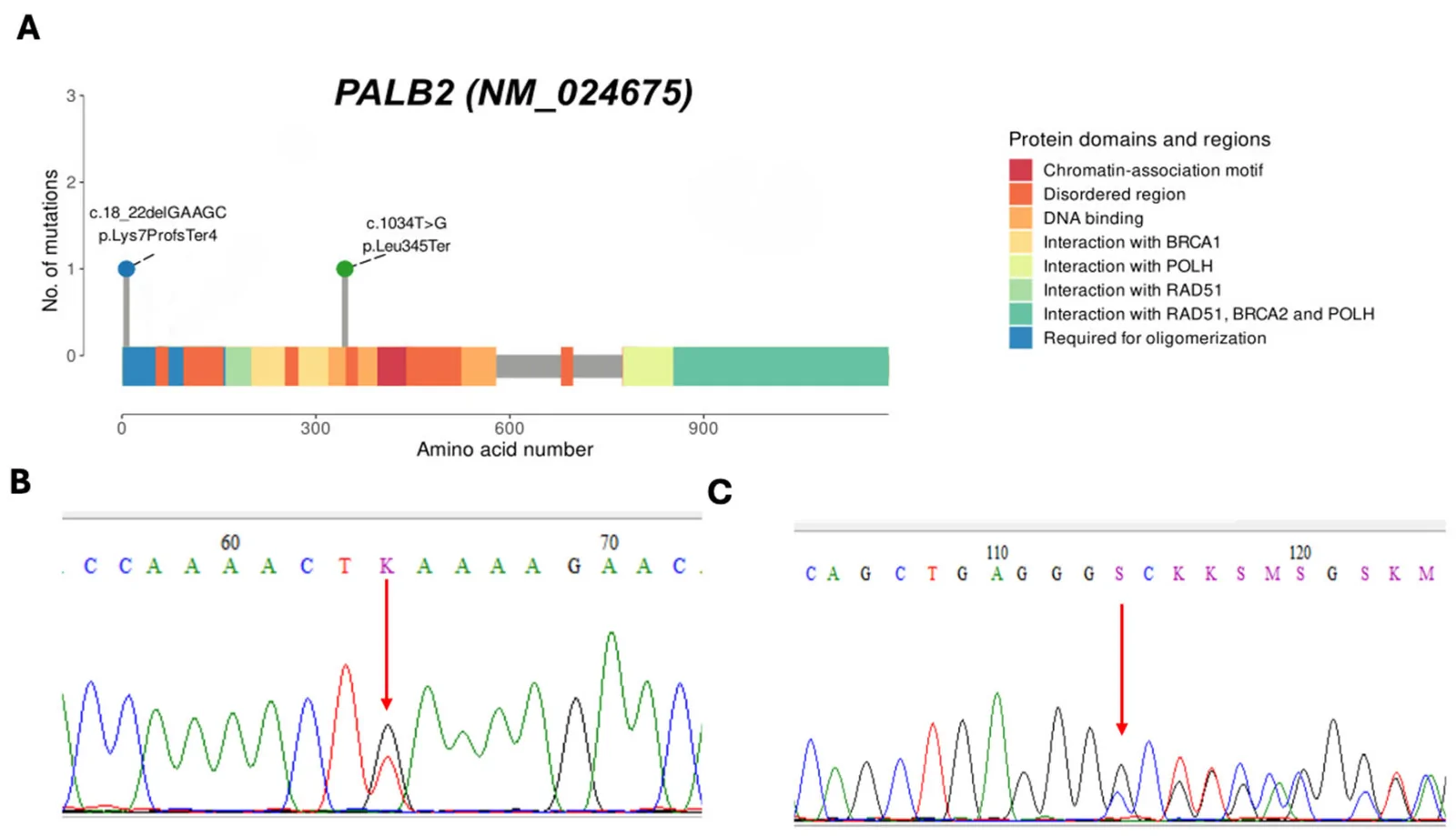
Identification of germline pathogenic variants in *PALB2* gene. (**A**) Lollipop plot showed the positions of both germline variants in protein domain region. (**B**) Sanger sequencing chromatogram showing the *PALB2* NM_024675.4:c.18_22delGAAGC frameshift variant identified in Case 1. (**C**) Sanger sequencing chromatogram showing the *PALB2* NM_024675.4:c.1034T>G nonsense variant identified in Case 2. Red arrows indicate the pathogenic variants.

Two women from Turkestan region of Kazakhstan with early-onset breast cancer were evaluated at the Kazakh Institute of Oncology and Radiology, Almaty. Neither patient reported a family history of breast cancer or other malignancies. Molecular genetic analysis of peripheral blood samples collected on 9 November 2017 identified two novel germline PALB2 pathogenic variants in the study patients (Figure 1A). According to the ACMG/AMP criteria, both variants were classified as pathogenic, supported by PVS1 (very strong; predicted loss-of-function in a gene where loss of function is an established disease mechanism).

Patient 1 carried the pathogenic variant NM_024675.4:c.18_22delGAAGC, a deletion located in exon 1 of the *PALB2* gene. This alteration results in a frameshift variant, disrupting the normal translational reading frame because the number of deleted nucleotides is not a multiple of three. Consequently, the variant is predicted to alter the downstream amino acid sequence and generate a truncated protein product (Figure 1B).

Patient 2 carried the pathogenic variant NM_024675.4:c.1034T>G, located in exon 4 of *PALB2*. This sequence alteration creates a premature termination codon (nonsense variant), leading to early termination of translation and production of a shortened protein transcript (Figure 1C).

Both variants are predicted to disrupt normal PALB2 protein function, potentially impairing homologous recombination-mediated DNA repair. Sanger sequencing chromatograms and PCR-RFLP analysis confirmed the variants identified by NGS.

### 3.2. Case 1: Pregnancy-Associated Locally Advanced Breast Cancer with Germline PALB2 c.18_22delGAAGC Variant

**Figure 2:**
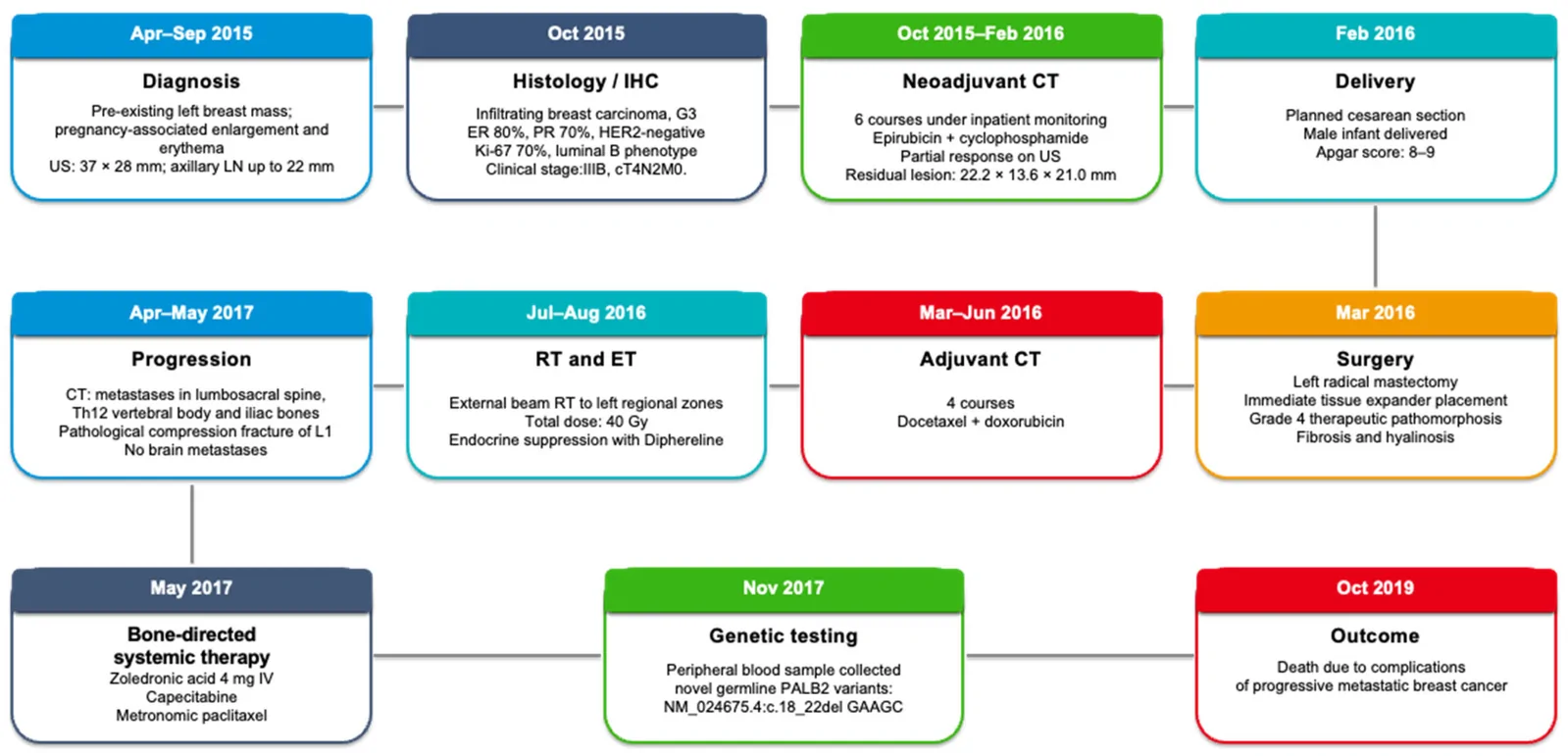
Clinical timeline of Case 1, illustrating the course from initial diagnosis and neoadjuvant chemotherapy to surgery, adjuvant treatment, metastatic progression, *PALB2* genetic testing, and final clinical outcome.

Case 1 was a 26-year-old pregnant woman who presented with progressive enlargement and erythema of a pre-existing mass in the left breast during pregnancy. Breast ultrasonography performed in September 2015 revealed a hypoechoic lesion measuring 37 × 28 mm, accompanied by left axillary lymphadenopathy measuring up to 22 mm and a chain of small left subclavian lymph nodes. A core needle biopsy performed in October 2015 confirmed grade 3 invasive ductal carcinoma (Figure 2).

**Figure 3:**
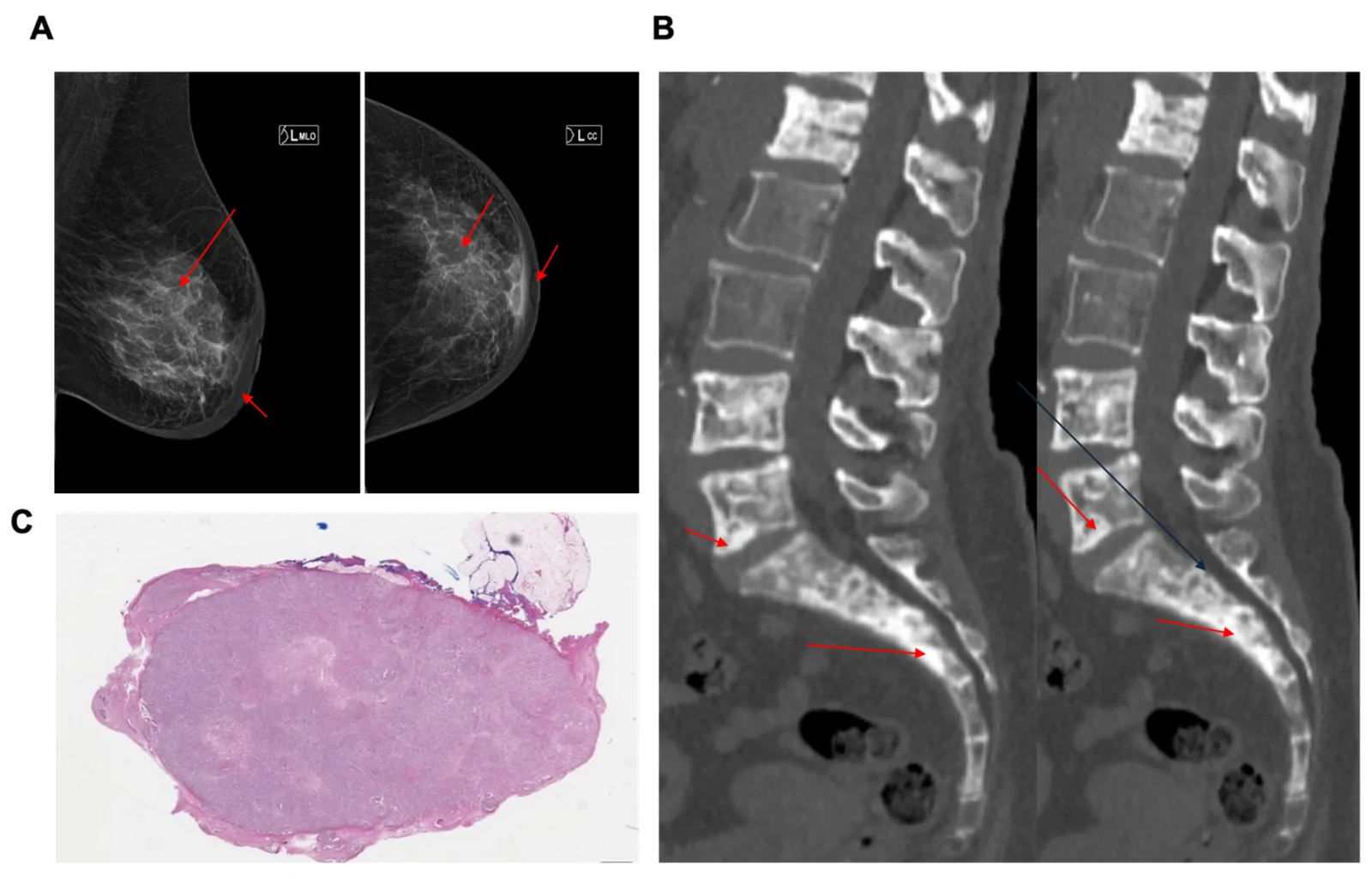
Clinical findings of Case 1. (**A**) Mammography of Patient1, after six cycles of neoadjuvant chemotherapy, obtained in craniocaudal and mediolateral oblique projections: diffuse moderate skin thickening, induration of the nipple–areolar complex, and nipple fixation are visualized on the left side. In the left breast, marked increased parenchymal density persists, with distortion of the stromal pattern due to thickened fibrous strands, as well as increased vascularity. (**B**) Lumbosacral spine CT scan findings are consistent with metastatic involvement of the lumbosacral spine, the T12 vertebral body, and the iliac bones. A pathological compression fracture of the L1 vertebral body is also identified. (**C**) Histological findings showing infiltrating breast carcinoma, grade 3, with an invasive growth pattern, prominent stromal desmoplasia, and extensive collagenized fibrotic areas within the tumor bed.

Immunohistochemical analysis demonstrated estrogen receptor positivity in approximately 80% of tumor cells, progesterone receptor positivity in approximately 70% of tumor cells, HER2 negativity, and a Ki-67 proliferation index of 70%. Based on these findings, the tumor was classified as luminal B, HER2-negative breast cancer. Clinically, the disease was staged as stage IIIB (cT4N2M0) with an edematous-infiltrative presentation (Figure 3A–C).

Following multidisciplinary team review, neoadjuvant chemotherapy was initiated under close inpatient monitoring. Between October 2015 and February 2016, the patient received six cycles of cyclophosphamide and epirubicin. Follow-up ultrasonography demonstrated a partial response, with reduction in the primary lesion to 22.2 × 13.6 × 21.0 mm and regression of axillary lymphadenopathy. On 11 February 2016, a planned cesarean section was successfully performed, resulting in the delivery of a healthy male infant with Apgar scores of 8 and 9.

Subsequently, on 5 March 2016, the patient underwent left radical mastectomy with immediate tissue-expander reconstruction. Histopathological examination revealed extensive treatment-related changes corresponding to grade 4 therapeutic pathomorphosis, characterized by marked stromal fibrosis and hyalinosis. Adjuvant treatment included docetaxel- and doxorubicin-based chemotherapy, radiotherapy, and endocrine therapy.

In March 2017, the patient developed persistent lower back pain. Computed tomography performed in April 2017 demonstrated metastatic involvement of the lumbosacral spine, Th12 vertebral body, and iliac bones, accompanied by a pathological compression fracture of the L1 vertebra (Figure 3B). Treatment for metastatic disease included zoledronic acid, capecitabine, and metronomic paclitaxel. Despite multimodal therapy, the disease continued to progress, and the patient died from metastatic breast cancer on 8 October 2019.

### 3.3. Case 2: De Novo Metastatic Breast Cancer with Germline PALB2 c.1034T>G Variant

**Figure 4:**
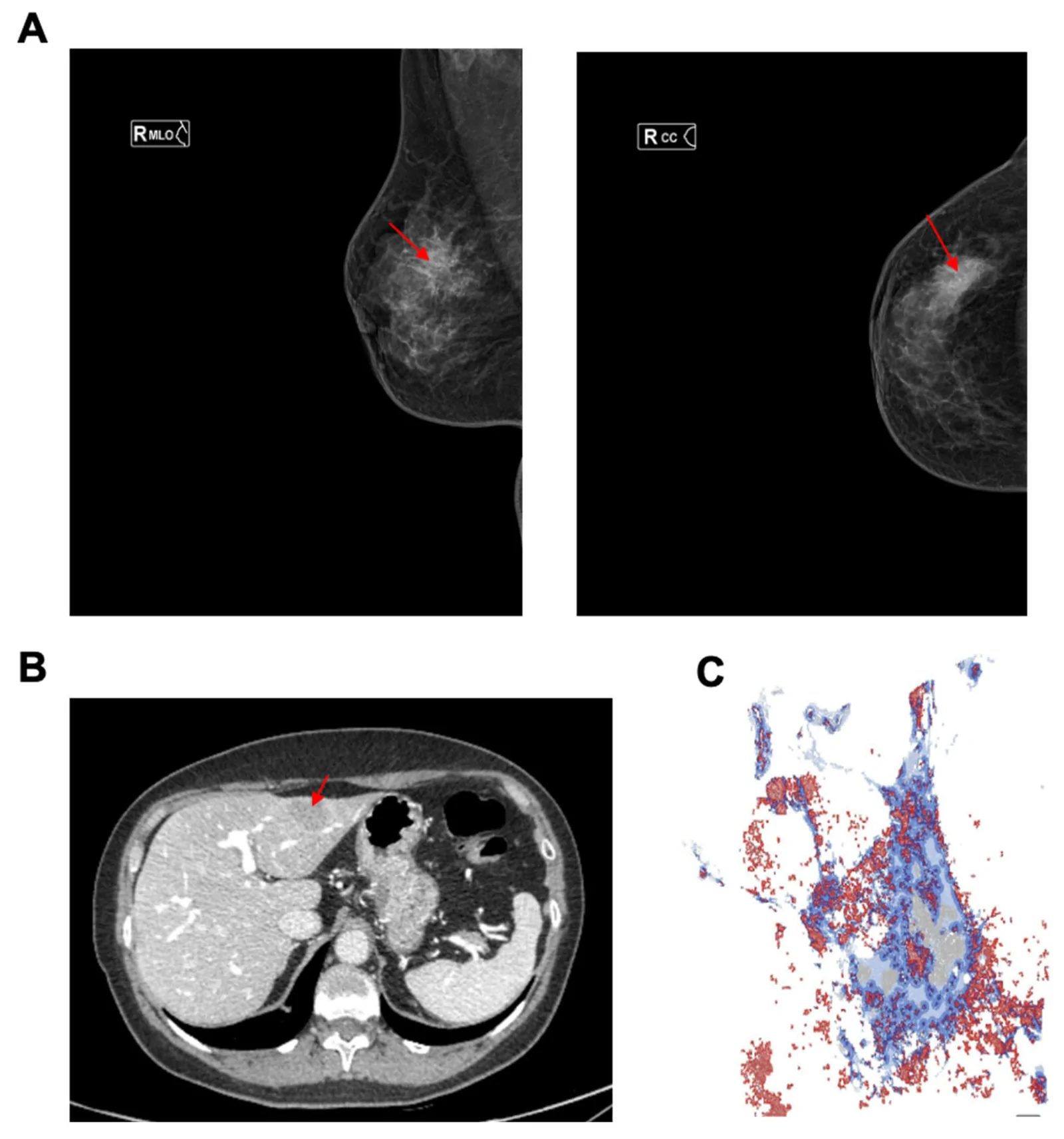
Clinical findings of Case 2. (**A**) Mammography of Case 2, dated 5 October 2017, performed before initiation of neoadjuvant chemotherapy and obtained in two standard projections, craniocaudal and mediolateral oblique: in the upper outer quadrant of the right breast, an irregular mass with indistinct, uneven margins and radiological features of infiltrative growth is visualized. Multiple fine microcalcifications are present within the lesion. Due to poorly defined borders, the exact extent is difficult to determine; the estimated size is approximately 4.5 × 4.0 × 3.0 cm. (**B**) Contrast-enhanced abdominal CT scan of Case 2 dated 12 October 2017: a poorly defined lesion measuring 20 × 21 × 13 mm is identified in segment III of the left hepatic lobe. The lesion demonstrates low native attenuation (+26 to +28 HU) with intense heterogeneous peripheral contrast enhancement up to +63 to +65 HU, radiologically suggestive of metastatic disease. A small calcification in the right hepatic lobe and mild retroperitoneal lymphadenopathy is also observed. (**C**) Histological findings of Case 2 dated 7 October 2017: examination of breast tissue core biopsy fragments reveals infiltrating breast carcinoma, grade 2, characterized by invasive tumor growth with foci of necrosis embedded in fibrotic and hyalinized stroma.

Case 2 was a 33-year-old woman who presented with a palpable mass in the right breast and was diagnosed with de novo metastatic breast cancer. Histopathological examination confirmed grade 2 invasive ductal carcinoma. Immunohistochemical analysis demonstrated strong estrogen receptor positivity, progesterone receptor positivity, HER2 negativity, and a Ki-67 proliferation index of 30%, consistent with luminal B, HER2-negative breast cancer (Figure 4A–C).

**Figure 5:**
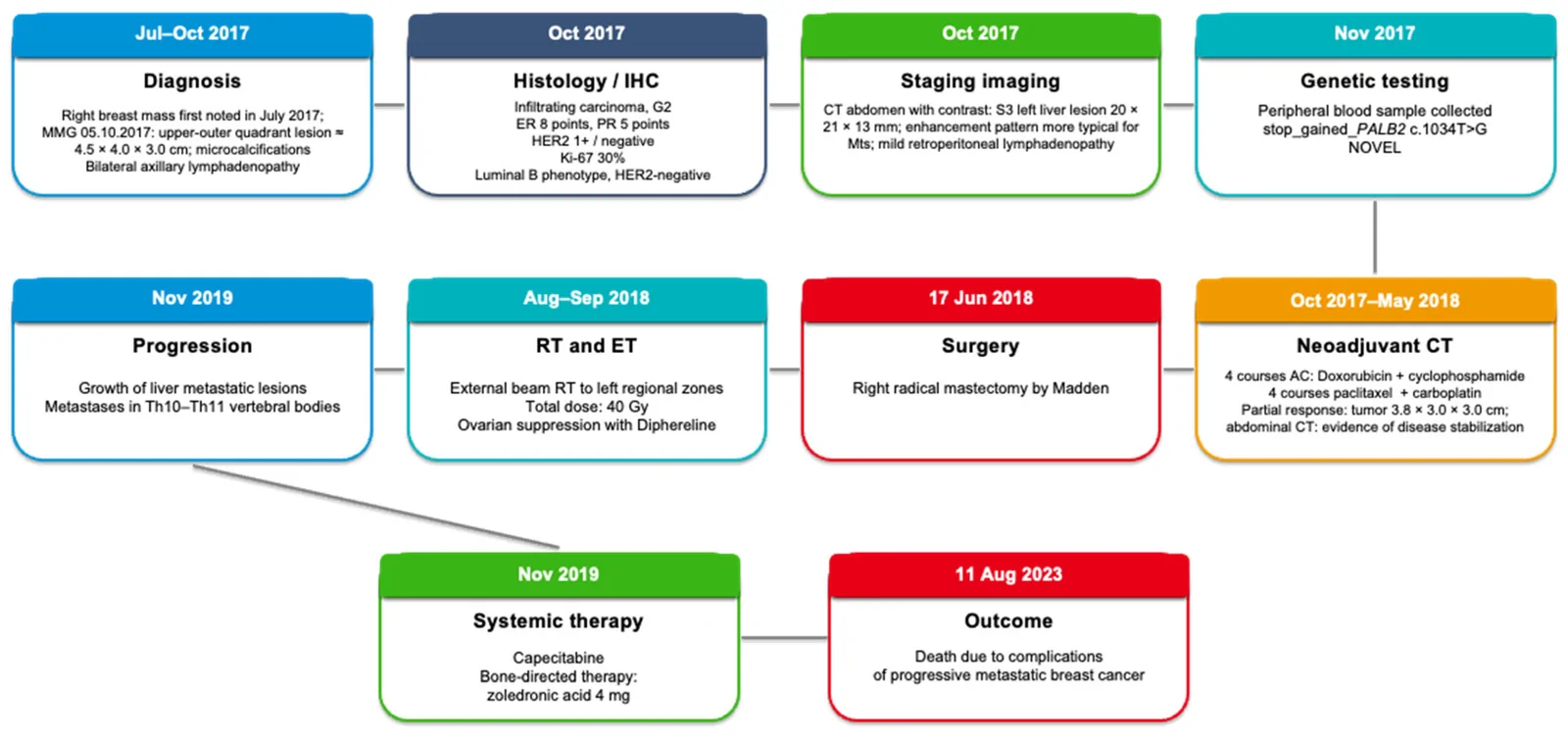
Clinical timeline of Case 2, summarizing the initial diagnosis, histological and immunohistochemical findings, staging imaging, neoadjuvant chemotherapy, surgery, adjuvant treatment, disease progression, genetic testing results, systemic therapy, and final clinical outcome.

Comprehensive clinical and radiological evaluation established a diagnosis of right-sided stage IV breast cancer (cT2N1M1, nodular form) with hepatic metastases at presentation. The patient initially received neoadjuvant chemotherapy consisting of doxorubicin and cyclophosphamide, followed by paclitaxel and carboplatin. This treatment resulted in partial regression of the primary breast lesion, while hepatic metastases remained radiologically stable. Following systemic therapy, she underwent right radical mastectomy according to Madden (Figure 5).

Adjuvant treatment included external beam radiotherapy to a total dose of 40 Gy and ovarian suppression with Diphereline. Despite this multimodal treatment approach, disease progression was subsequently observed, characterized by enlargement of hepatic metastatic lesions and development of bone metastases involving the Th10 and Th11 vertebral bodies. Additional systemic treatment with capecitabine and bone-targeted therapy with zoledronic acid was administered; however, the disease continued to progress. The patient ultimately died from complications of metastatic breast cancer.

### 3.4. Comparative Clinical Characteristics and Outcomes

Both patients developed breast cancer at a young age and had no reported family history of breast or other malignant tumors. Germline *PALB2* variants were identified in both cases, suggesting a hereditary predisposition despite apparently sporadic clinical presentations. Histologically, both tumors were invasive ductal carcinomas with a luminal B, HER2-negative molecular subtype.

Although their initial presentations differed, both patients experienced aggressive clinical courses and poor outcomes. Patient 1 presented with pregnancy-associated, locally advanced stage IIIB breast cancer and subsequently developed extensive skeletal metastases. In contrast, Patient 2 presented with de novo stage IV disease with hepatic metastases and later developed additional osseous involvement. Despite receiving comprehensive multimodal treatment, including chemotherapy, surgery, radiotherapy, endocrine therapy, and treatment for metastatic disease, both patients experienced progressive metastatic spread and ultimately died from breast cancer-related complications.

## 4. Discussion

The identification of a germline pathogenic *PALB2* variant has substantial clinical significance, as it enables a shift from a standard breast cancer management approach to a risk-adapted strategy. In the international study by Yang et al., pathogenic *PALB2* variants were associated with a high risk of breast cancer, as well as moderately increased risks of ovarian and pancreatic cancers [19]. Therefore, early genetic testing is particularly important in young patients, patients with a positive family history, those with an aggressive disease course, or cases with suspected hereditary tumor predisposition. Determination of *PALB2* status before surgery allows clinicians to discuss the extent of surgical intervention, the risk of a second primary breast cancer, and an individualized follow-up plan in advance. If *PALB2* status is established after surgery, it may still influence subsequent management by supporting intensified surveillance of the remaining breast tissue, particularly with breast MRI.

The presence of a *PALB2* variant is not an absolute indication for bilateral mastectomy. Masanam et al. emphasized that contralateral risk-reducing mastectomy should be considered on an individual basis, taking into account patient age, family history, tumor phenotype, the availability of regular surveillance, and patient preferences [20]. This approach is consistent with the findings of Yadav et al., who demonstrated that the risk of contralateral breast cancer among *PALB2* carriers is heterogeneous and appears to be particularly increased in patients with estrogen receptor-negative primary breast cancer [21]. Thus, preservation of the contralateral breast may be an appropriate strategy in selected patients, provided that active surveillance is feasible. In this context, annual breast MRI combined with mammography represents an important tool for the early detection of malignant changes. Lowry et al. demonstrated that MRI-based screening in *PALB2* carriers may reduce breast cancer mortality [22].

The issue of prophylactic removal of the ovaries and fallopian tubes should also be approached individually. According to the ACMG recommendations, the NCCN Clinical Practice Guidelines, and the UK consensus guidelines, risk-reducing salpingo-oophorectomy is not mandatory for all PALB2 carriers; rather, it may be discussed around 45–50 years of age, particularly in the presence of a strong family history of ovarian cancer [23,24].

Although PALB2 loss provides a biological rationale for sensitivity to DNA-damaging therapy and PARP inhibition, routine adjuvant PARP inhibitor therapy for early-stage PALB2-associated breast cancer remains investigational and has not yet been established as standard care [25,26]. The strongest clinical evidence for PARP inhibition in PALB2-associated breast cancer is currently available in the metastatic setting. In the TBCRC 048 phase II trial, olaparib demonstrated substantial activity in metastatic breast cancer with germline PALB2 pathogenic variants, with high response rates and clinically meaningful progression-free survival [25]. More recent expansion cohort data confirmed this activity, reporting an objective response rate of 75%, a clinical benefit rate of 83.3%, and a median progression-free survival of 9.4 months in patients with germline PALB2-mutated metastatic breast cancer [26].

PALB2 status alone should not automatically determine the use of platinum-based chemotherapy in every patient. Current evidence supporting platinum sensitivity is based mainly on biological rationale, case series, and extrapolation from homologous recombination deficiency, rather than large PALB2-specific randomized trials [27]. Therefore, platinum therapy may be most appropriate in clinical scenarios in which its use is already supported by breast cancer treatment standards, such as aggressive HER2-negative disease, triple-negative breast cancer, visceral crisis, or metastatic disease requiring a rapid tumor response.

### Limitations

Several limitations of this study must be acknowledged. Foremost, this is a descriptive case report based on a cohort of only two patients (n = 2). Given this small sample size, our findings are inherently hypothesis-generating and observational in nature; they cannot be generalized to the broader population of *PALB2* variant carriers or to patients with hereditary breast and ovarian cancer syndrome.

## 5. Conclusions

We report two novel *PALB2* variants in patients with breast cancer: a nonsense variant introducing a premature termination codon and a truncating frameshift deletion, both predicted to disrupt critical interactions of PALB2 with BRCA1 and BRCA2. These findings expand the spectrum of *PALB2* variants and underscore the need for functional assays and family segregation studies to clarify the clinical significance of newly identified *PALB2* variants. Identification of such variants has implications for genetic counseling, risk-reduction strategies, and potential treatment decisions involving DNA-damage–targeted therapies.

## Figures and Tables

**Table 1:** PCR amplification conditions for direct sequencing.

No.	Genetic Variant	Primer Sequence (5′→3′)	Primer Annealing Temperature	PCR Product Size (bp)
1.	*PALB2 c.1034T>G*	f:GCCAACTGCCCACAAGTTCT r:GAGGAGAGGTTGCTTCCAGG	58 °C	228
2.	*PALB2-c.18_22delGAAGC*	f:CTTTTCGTTCTGTCGCCTGC r:TAAGAGGAGGGGGTGGTCAG	56 °C	194

**Table 2:** Primer sequences and restriction enzymes for genotyping of *PALB2* gene variants.

Genetic Variant	Primer Sequence (5′→3′)	PCR Product Size (bp)	Restriction Enzyme	Amplicon Restriction Sizes (bp)
*PALB2 c.1034T>G*	f:GCCAACTGCCCACAAGTTCT r:GAGGAGAGGTTGCTTCCAGG	228	*MseI*	With mutation (heterozygote): 228, 132, and 96 Without mutation: 228
*PALB2 c.18_22delGAAGC*	f:CTTTTCGTTCTGTCGCCTGC r:TAAGAGGAGGGGGTGGTCAG	194	*AvaI*	With mutation: 194 Without mutation (heterozygote): 194, 158 and 36

## Data Availability

The datasets generated and/or analyzed during the current study are available from the corresponding author upon reasonable request.
